# Burden of respiratory syncytial virus diseases among under 5 children in Sub-Saharan Africa: *A systematic review and meta-analysis*

**DOI:** 10.1016/j.heliyon.2023.e22211

**Published:** 2023-11-19

**Authors:** Fiseha Wadilo Wada, Minyahil Tadesse Boltena, Rawliegh Howe, Fithamlak Bistegen Solomon, Adey Feleke, Tamrayehu Seyoum, Andargachew Mulu, Adane Mihret

**Affiliations:** a Armauer Hansen Research Institute, Ministry of Health, Ethiopia; b Department of Biomedical Sciences, College of Natural and Computational Sciences, Addis Ababa University, Addis Ababa, Ethiopia; c Department of Microbiology, Immunology, And Parasitology, School of Health Sciences, Addis Ababa University, Addis Ababa, Ethiopia; d Department of Medical Laboratory Sciences, College of Health Sciences and Medicine, Wolaita Sodo University, Ethiopia; e Evidence Based Health Care, Ethiopian-Evidence Based Health Care Centre: A JBI Center of Excellence, Public Health Faculty, Institute of Health, Jimma University, Jimma, Ethiopia

**Keywords:** Respiratory syncytial virus, ALRIs, Children, Naso/oropharyngeal, Sub-Saharan Africa

## Abstract

**Background:**

Respiratory syncytial virus (RSV) is the most common cause of acute lower respiratory infections (ALRIs) in young children. To design preventive efforts in sub-Saharan Africa, a better knowledge of the true role of RSV in pediatric ALRIs is required. Therefore we conducted a systematic review and meta-analysis of case–control studies to estimate the etiological role of RSV to ALRIs in under 5 years children in sub-Saharan Africa.

**Methods:**

This study was done according to PRISMA guidelines. PubMed, EMBASE, SCOPUS, Web of Sciences databases, and Google Scholar were used to retrieve articles. STATA software version 17 was used for data analysis. The results of all the included studies were standardized to odds ratios (ORs) with accompanying 95 % confidence intervals (95 % CIs) and the pooled estimates of ORs, attributable fraction among the exposed (AFE), and population attributable fraction (PAF) were reported. The heterogeneity was assessed using Cochrane chi-square (I 2) statistics.

**Result:**

A total of 6200 cases and 4986 controls from 14 articles that fulfilled the inclusion criteria were included. The pooled prevalence of RSV among cases and controls was 23.52 % [95 % CI (20.68–26.47)] and 4.33 % [95 % CI (3.11–5.73)], respectively. The pooled OR is 7.04 [95 % CI (4.41–11.24)], which indicated a significant association between RSV and ALRI. Among ALRIs cases positive for RSV, the proportion of disease that was not attributable to the background rate (AFE) was 85.8 % [95 % CI (77.3–91.1)]. The fraction of ALRIs children that can be attributed to RSV (PAF) was 20.2 % [95 % CI (16–24.1)].

**Conclusion:**

This study showed clear associations between RSV and ALRI hospitalization in young children in sub-Saharan Africa indicating the need for prophylactic measures against RSV in this age group.

## 1 Background

Acute lower respiratory infections (ALRIs) of any origin is the leading cause of mortality and morbidity among under 5 children [1]. Respiratory syncytial virus (RSV) is the most common cause of ALRIs in young children, which contributes substantially to morbidity and mortality. One in every 50 deaths in children aged 0–60 months and one in every 28 deaths in children aged 28 days to 6 months is attributable to RSV [2]. RSV is a negative-sense, single-stranded RNA virus that belongs to the Pneumoviridae family and has two major sub-types: type A and type B which share disease manifestations and 95 % sequence identity [3,4].

RSV has been a priority for vaccine and anti-viral development for close to 6 decades [5,6] but yet there is no licensed vaccine against RSV infection. However, a single dose of RSV prefusion F protein–based vaccine candidate showed 82.6 % efficacy against RT-PCR–confirmed RSV-related lower respiratory tract disease in older children in an ongoing international Phase 3 trial. The efficacy was similar against the RSV A and B subtypes [7]. Furthermore, there is a lack of available treatment options, which are mostly limited to supportive care. However, for young children at high risk of serious RSV disease, immune prophylaxis initially with high RSV antibody and later a neutralizing monoclonal antibody, palivizumab, is effective and available [5].

In addition, RSV diagnostic testing is challenging in resource limited sub-Saharan African countries. The sensitivity of antigen-based testing is low and the more sensitive polymerase chain reaction (PCR)-based testing is almost nonexistent due to its relatively high costs. Detection of RSV infections is further complicated by the lack of uniform clinical case definition and the non-specificity of its symptoms and the obvious challenge to distinguish colonization from infection. Thus, estimating the true burden of acute lower respiratory infections (ALRIs) due to RSV is very challenging particularly in resource limited settings. To resolve the latter, several studies use case-control study design and compare the infection status of people with ALRIs (cases) to people without ALRIs (controls) [[8], [9], [10], [11], [12], [13], [14]]. Consecutively, there is underestimation of the RSV disease burden in developing countries despite the growing body of evidence indicating that it may be comparable to the diseases burden of influenza.

Thus, a better understanding of the real contribution of RSV in childhood ALRIs is needed to guide clinical management and preventive measures in sub-Saharan Africa particularly when promising RSV candidate vaccines or therapy are currently under evaluation. Therefore, we conducted a systematic review and meta-analysis of case–control studies aiming at estimating the role of RSV in the etiology of ALRIs in under 5-year-old children in sub-Saharan Africa.

## 2 Methods

### 2.1 Protocol registration

The study protocol has been registered in the International Prospective Register of Systematic Reviews (PROSPERO) with registration code CRD42022361757 [15].

### 2.2 Search strategy and selection criteria

**Fig. 1:**
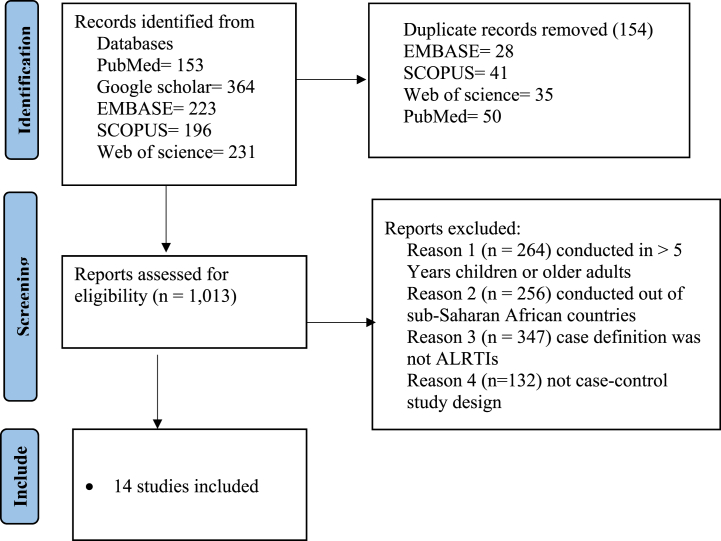
PRISMA flow diagram showing the selection process of eligible articles for systematic review and meta-analysis.

We conducted and reported a systematic review and meta-analysis according to the Preferred Reporting Items for Systematic Reviews and Meta-analyses (PRISMA) flow diagram (Fig. 1) [16]. The combination of MeSH/Emtree terms and free text words were used to run for each database using Boolean operators “AND” and “OR”. EMBASE, PubMed, Scopus, Web of Science databases and Google Scholar were used to retrieve the studies (Supplementary table 1). The reference lists of all included studies were screened to obtain additional studies and authors were contacted to receive any missing articles. EndNote version 20.2.1. was used to remove duplicates. Two independent reviewers (Fiseha Wadilo and Tamrayehu Seyoum) screened titles and abstracts.

Potentially relevant studies were retrieved in full text from February 7–14, 2023 and eligible studies were assessed in detail against the inclusion criteria by two reviewers (FisehaWadilo and Tamrayehu Seyoum). Reasons for the exclusion of studies during full text critical appraisal were recorded and reported. Discrepancies between reviewers during screening at each stage were resolved through discussion.

**Inclusion criteria:** We included studies that fulfilled our strict eligibility criteria: studies in children younger than five years of age; studies investigating etiological role of RSV using RT PCR; studies conducted in sub-Saharan African countries, studies where respiratory specimens were collected and diagnostic test conducted using qRT PCR; case–control/prospective cohort studies that reported data in both case and control groups; studies of under 5-year-old children with clinical pneumonia (or lower respiratory infection); studies that have conducted on all age groups and disaggregated by age category and data on under 5 is available, and case control studies reporting virus–specific proportions separately in both groups. We only included studies where the case definition for ALRI (or clinical pneumonia) was clearly defined and consistently applied.

**Exclusion criteria:** We excluded studies that were conducted outside sub-Saharan Africa. We also excluded studies conducted in children >5 years of age, those using cross-sectional study design, those not using valid laboratory diagnostic tests, and those without clear case and control definition.

### 2.3 Data extraction

Data was extracted to an excel spreadsheet. A data extraction tool was prepared that included author (s) name, publication year, study design, sample size, and study area, case and control definition, type of respiratory specimens used, RNA extraction techniques, diagnostic assay, total number of pathogens isolated in cases and controls. Data extraction was conducted by FW and TS. In addition, there were two rounds of meetings for further data cross-check and validation.

### 2.4 Data quality and risk of bias assessment

FW and TS did an assessment of the methodological quality of eligible studies using the Joanna Briggs Institute's critical appraisal instrument for prevalence studies (Supplementary Table 2). The results of the critical appraisal were reported in narrative form and a table. Lower risk of bias (95 %) was observed after the assessment. Articles were reviewed using titles, abstracts, and full text screening.

### 2.5 Statistical analysis

Data synthesis and statistical analysis were conducted using STATA version 17 software. The results of all the included studies were standardized to odds ratios (ORs) with accompanying 95 % confidence intervals (95 % CIs) to facilitate interpretation and analysis in the subsequent meta–analyses. Whenever possible, we extracted adjusted odds ratios (aOR). We performed the meta–analysis of case-control studies and reported pooled estimates of ORs with corresponding 95 % CIs using the random effects model (DerSimonian–Laird method) because these studies do not share common effect size due to methodological heterogeneity [17]. The heterogeneity was assessed using Cochrane chi-square (I^2^) statistics, while Egger intercept was used to assess publication bias. The p-value of <0.05 for I^2^ statistics was used to determine the presence of heterogeneity. The findings were reported using the pooled ORs with a 95 % confidence interval (CI) and forest plot.

The virus–specific attributable fraction among the exposed (AFE) was used to explore the etiological role of RSV in ALRI patients. Among ALRI cases positive for RSV, the proportion of ALRI that is not attributable to the background rate is AFE = 1-(1/OR). From the total ALRI cases, the fraction of ALRI cases that can be attributed to RSV (PAF) was also estimated. The following formula was applied: *PAF = P* (*E \ M*) *x* (*1-*(*1/OR*)); Where *PAF* is the population attributable fraction*; P* (*E \ M*)*,* the pooled prevalence of RSV in ALRI cases*;* and *OR,* the pooled odds ratio.

## 3 Result

### 3.1 Characteristics of included studies

The electronic databases search identified 1167 articles, from which 154 articles were removed due to duplication. After reading the title and abstract, only 14 articles that fulfilled the inclusion criteria were included in this systematic review and meta-analysis. The selection of articles is reflected in Fig. 1.

Studies included in this meta-analysis were published between 2013 and 2021. Most of the studies were conducted in a hospital setting. The characteristics of included articles are presented in Table 1. Most of the studies were conducted in Kenya (n = 6). All of the studies were used a case-control study design and were done in children <5 years of age. The case and control definitions used by each included article are presented in Table 1.

**Table 1:** Study characteristics.

Authors, Year	Country	Study Period	Age	Study design	Healthcare set up	Case definition	Control definition
Kelly et al., 2015 [18]	Botswana	April 2012 and August 2014	1–23 Months	prospective cohort and case-control	Hospital	WHO pneumonia definition	Children without pneumonia matched to cases by primary care clinic and date of enrollment
Bigogo et al., 2013 [19]	Kenya	March 2007 and February 2011	<5	Case-control	population-based infectious disease surveillance	WHO pneumonia definition	Presented to a study health facility with non-severe illness
Fuller et al., 2013 [20]	Kenya	August 1, 2008 to December 31, 2010,	<5	Case-control	Hospital	Inpatients with SARD, ILI, or respiratory symptoms	Afebrile outpatients with no respiratory or gastrointestinal symptoms in previous 2 weeks.
Bénet et al., 2015 [21]	Mali	July 2011–December 2012	<5	A Prospective Case-Control Study	Hospital	WHO pneumonia definition	Hospitalized children without respiratory features, matched for age and period
Zar et al., 2016 [22]	South Africa	May 29, 2012, to Dec 1, 2014	<42 M	nested case-control	Hospital	any episode of pneumonia, excluding congenital pneumonia	Asymptomatic controls and with mild symptoms of upper respiratory tract infection. Controls were matched to cases by birth date (to within 2 weeks), age of presentation (to within 2 weeks), and site of enrolment.
Hammitt et al., 2012 [23]	Kenya	January–December 2010	1–59 months	Case-control	Hospital	WHO, severe pneumonia (SP) or very severe pneumonia (VSP).	Who did not meet the case definition for SP or VSP and were recruited using marginal frequency matching by age group and month of year.
Mwananyanda et al., 2021 [24]	Zambia	November 2011 and October 2013	1–59 Months	Case-control	Hospital	PERCH study Case definition(i.e. WHO-defined severe or very severe pneumonia (pre-2013 definitions, originally presented in 2005))	PERCH study control definition (i.e. randomly selected from residents of the same catchment area as cases and frequency matched to cases by age group (1 to <6 months, 6 to <12 months, 12 to <24 months, and 24–59 months of age))
Tapia et al., 2021 [25]	Mali	January 1, 2012, and January 14, 2014	28 days to 59 months	Case-control	Hospital		
Awori et al., 2021 [26]	Kenya	August 15, 2011, to November 15, 2013	28 days to 59 months	Case-Control	Hospital		
Howie et al., 2021 [27]	Gambia	November 3, 2011 and November 2, 2013	1–59 months	Case-Control	government health centers		
Moore et al., 2021 [28]	South Africa	Between August 17, 2011, and September 4, 2013	1–59 months	Case-Control	Hospital		
Bénet et al., 2017 [29]	Madagascar	May 2010 to June 2014	2–60 months	case-control study	Hospital	Pneumonia cases as defined by the WHO	no signs/symptoms of respiratory illness/URTI; were hospitalized for surgery or attending routine outpatient appointment (mild illnesses, routine monitoring, immunization, etc) at the hospital site
Feikin et al., 2013 [8]	Kenya	January 1, 2009–February 28, 2010	<5 years	Case-Control	Hospital	SARI (WHO-defined severe or very severe pneumonia, or oxygen saturation <90 %)	Presented with no severe illness, for immunizations, or for medicine refills. Eligible controls could not have had fever, any respiratory symptoms or diarrhea during the preceding two weeks.
Breiman et al., 2015 [30]	Kenya	March 1, 2007–February 28, 2011	<5 years	Surveillance/community-based case-control	Household surveillance		

### 3.2 Characteristics of the study population

A total of 6200 cases and 4986 controls were counted for this meta-analysis (Table 2). The highest sample size for both cases and controls was used by Moore et al. [28], which were 795 and 823, respectively. Most of the studies summarized the age of the children using median (IQR) and were reported in months. The minimum median age reported for both cases and controls was 4 months. In contrast, the maximum median age reported for cases and controls was 18 and 21.6, respectively.

**Table 2:** Characteristics of study population.

Authors, Year	Cases (n = 6200)	Controls (n = 4986)	Cases Median (IQR) age(Month)	Control Median (IQR) age(Month)
Kelly et al., 2015 [18]	310	133	7.0 (3.0–13.3)	6.4 (4.0–12.2)
Bigogo et al., 2013 [19]	538	193	Not mentioned	Not mentioned
Fuller et al., 2013 [20]	680	136	Not mentioned	Not mentioned
Bénet et al., 2015 [21]	118	98	12 (5–26)	11 (5–23)
Zar et al., 2016 [22]	284	412	5 (3–9)	5 (2–8)
Hammitt et al., 2012 [23]	105	190	9.7 (3.8–18.7), 13 (average)	20 (average)
Mwananyanda et al., 2021 [24]	473	530	4.0 (3.0, 5.0)	4.0 (2.0, 4.0)
Tapia et al., 2021 [25]	650	724	6 (3–13)	9 (4–20)
Awori et al., 2021 [26]	628	855	Not mentioned	Not mentioned
Howie et al., 2021 [27]	609	624	8 (3–18)	11 (5–22)
Moore et al., 2021 [28]	795	823	5.0 (2.0–12.0)	8.0 (4.0–16.0)
Bénet et al., 2017 [29]	80	60	mean (SD),22.2 (14.6)	mean (SD),17.1 (12.6)
Feikin et al., 2013 [8]	199	93	18	15
Breiman et al., 2015 [30]	731	115	18	21.6

### 3.3 Sample and laboratory methods characteristics

Most of the studies collected NP/OP swabs sample and the detection of RSV was done using multiplex PCR (Table 3). The most commonly used assay platform was Fast-track Diagnostics, Luxembourg.

**Table 3:** Sample and Laboratory Methods characteristics.

Authors, Year	sample type	Extractions	Diagnostic assay	Assay platform	Target pathogens
Kelly et al., 2015 [18]	NP swab	Not mentioned	real-time multiplex PCR and uniplex PCR assay	Not mentioned	Influenza viruses, Influenza A, Influenza B, PIV, PIV1, PIV2, PIV3, Human metapneumovirus, Adenovirus, Rhinovirus/enterovirus, Rhinovirus A, Rhinovirus B, Rhinovirus C, Rhinovirus other
Bigogo et al., 2013 [19]	NP/OP swabs	MagMax viral RNA kit and Kingfisher mL instrument (Life Technologies, New York, NY)	AgPath-ID One-step RT-PCR kit	Applied Biosystems, Foster City, CA)	RSV
Fuller et al., 2013 [20]	NP/OP swabs	QIAamp Viral RNA Minikit (Qiagen, Valencia, CA)	AgPath-ID One- Step RT-PCR Reagents	Applied Biosystems, Foster City, CA)	Adenovirus, RSV, Human metapneumovirus, Influenza, PIV
Bénet et al., 2015 [21]	nasal swabs and pleural effusions	Not mentioned	FTD respiratory pathogens 21 plus	Fast-track Diagnostics, Luxembourg	*S. pneumoniae, S. aureus* *H. influenza*, Mycoplasma spp., Chlamydia spp., Human metapneumovirus, Coronavirus NL63, Coronavirus 229E, Coronavirus OC43, Coronavirus HKU 1, Adenovirus, Enterovirus, Parechovirus, Rhinovirus, RSV, PIV1, PIV2, PIV3, PIV4, Influenza A, Influenza B, Influenza A(H1N1), Bocavirus
Bénet et al., 2017 [29]	NP swabs				
Hammitt et al., 2012 [23]	NP/OP swab and induced sputum (IS)	Not mentioned	multiplex PCR for 16 respiratory pathogens	Not mentioned	RSV, Adenovirus, PIV, Influenza A, Influenza B *B. pertussis, M. pneumoniae*
Zar et al., 2016 [22]	2 NP swabs	QIAsymphony Virus/Bacteria Mini Kit (Qiagen, Hilden, Germany).	33-pathogen multiplex quantitative PCR	FTD Resp-33; Fast-track Diagnostics, Sliema, Malta	*B. pertussis, C. pneumoniae* *H. influenzae* type b Non-type b *H. influenzae* *M. catarrhalis, M. pneumoniae* *S. pneumoniae,* Salmonella spp S. aureus, P. jirovecii Adenovirus, Human, cytomegalovirus, Coronavirus 229, Coronavirus 43, Coronavirus 63, Coronavirus HKU, Influenza A, Influenza B, Influenza C, Human bocavirus, Human metapneumovirus A/B, PIV1, PIV2, PIV3, PIV4, Parechovirus/Enterovirus, Human rhinovirus, RSV
Mwananyanda et al., 2021 [24]	NP/OP swabs	NucliSENS easyMAG platform (bioMérieux, Marcy l’Etoile, France)			
Tapia et al., 2021 [25]	NP/OP swabs				
Awori et al., 2021 [26]	NP/OP swabs				
Howie et al., 2021 [27]	NP/OP swabs				
Moore et al., 2021 [28]	NP/OP swabs				
Feikin et al., 2013 [8]	NP/OP swabs	Qiagen's QIAamp viral RNA and MagMAX Viral RNA Isolation Kit (Applied Biosystems) mini kit (Qiagen Inc, Valencia, CA)	TaqMan Universal PCR Master Mix, and AgPath-ID One-Step RT-PCR Reagents	Applied Biosystems	Influenza A Influenza B Influenza A or B RSV Adenovirus PIV1, PIV2, PIV3, Human metapneumovirus Mycoplasma pneumoniae Rhinovirus/enterovirus
Breiman et al., 2015 [30]	NP/OP swabs and three mL of blood for culture	MagMAX Viral RNA Isolation Kit (Applied Biosystems)	qRT-PCR	Not mentioned	Influenza A, Influenza B, Influenza A or B, RSV, Adenovirus, PIV1, PIV2, PIV3, Any parainfluenza, Human, metapneumovirus, Rhinovirus/Enterovirus, Parechovirus

Respiratory syncytial virus, RSV; Parainfluenza virus 1, PIV 1; Parainfluenza virus 2, PIV 2; Parainfluenza virus 3, PIV 3; Parainfluenza virus 4, PIV 4.

### 3.4 The pooled prevalence of RSV among cases and controls

**Fig. 2:**
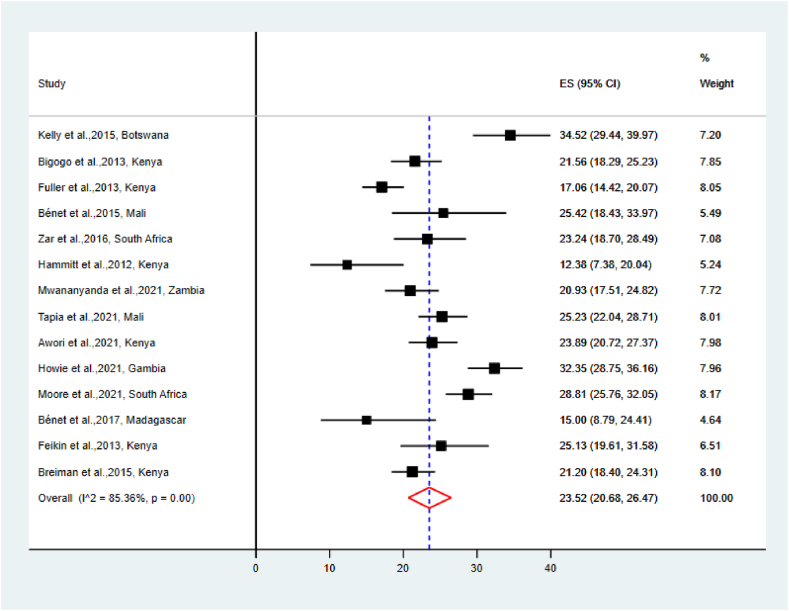
The Pooled prevalence of RSV among cases.

**Fig. 3:**
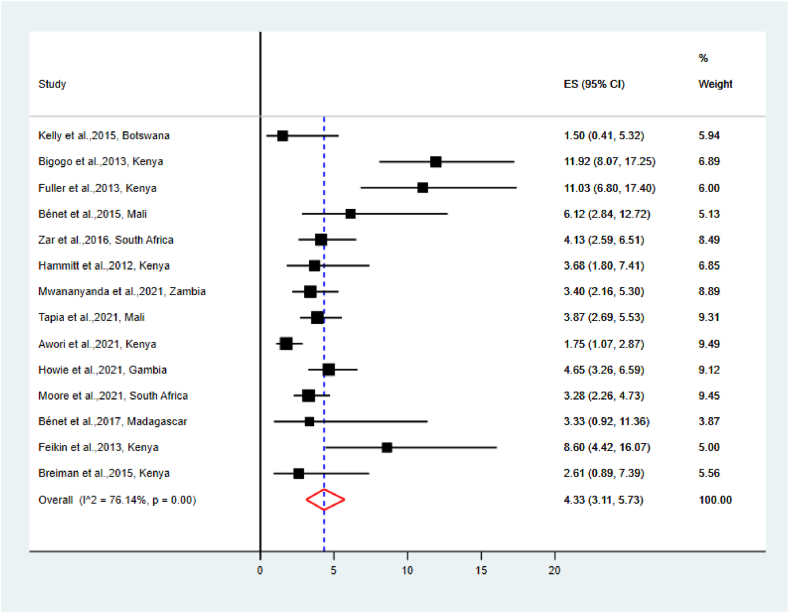
The Pooled prevalence of RSV among controls.

The pooled prevalence of RSV among cases and controls was 23.52 % [95 % CI (20.68–26.47)] and 4.33 % [95 % CI (3.11–5.73)], respectively (Fig. 2, Fig. 3). The highest reported prevalence of RSV among cases and controls was 34.52 % and 11.92 %, respectively; conversely, the lowest reported prevalence of RSV among cases and controls was 12.38 % and 1.50 %, respectively.

### 3.5 RSV pooled OR, AFE and PAF

**Fig. 4:**
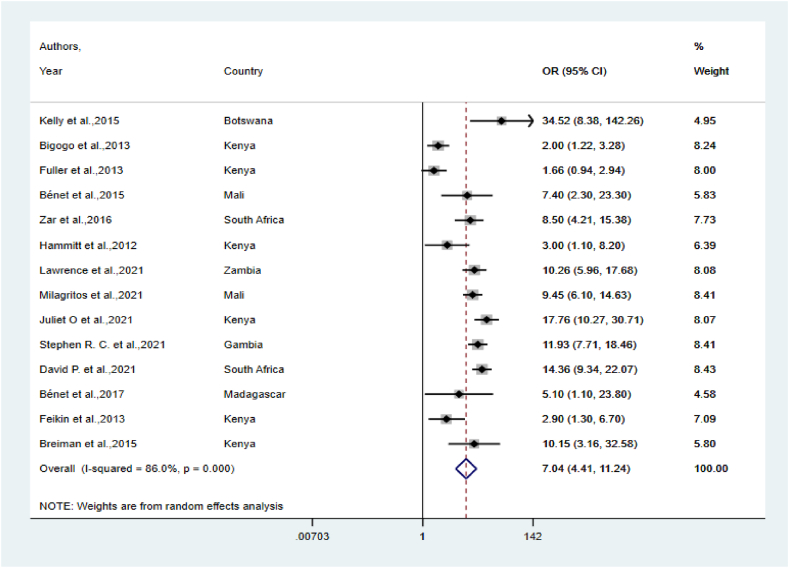
RSV pooled estimates of ORs with corresponding 95 % CIs using the random effects model.

Meta-analyses of RSV OR were reported as well as the corresponding attributable fraction among the exposed (AFE) and population attributable fraction (PAF) (Fig. 4). The pooled OR was 7.04 [95 % CI (4.41–11.24)], indicating a significant association between RSV and ALRIs in children in sub-Saharan Africa. Among ALRIs cases positive for RSV, the proportion of disease that was not attributable to the background rate (AFE) was 85.8 % [95 % CI (77.3–91.1)]; this shows clear associations between this virus and ALRI hospitalization in young children. The fraction of ALRI child cases that can be attributed to RSV (PAF) was 20.2 % [95 % CI (16–24.1)]. Therefore, this indicates the potential for substantive reductions in the number of ALRI cases in young children using preventive and/or prophylactic measures such as vaccination.

### 3.6 Other respiratory viruses associated with ALRIs

Acute lower respiratory infections were also significantly associated with other respiratory

viruses, including Influenza A virus, Parainfluenza virus, and Metapneumovirus. (Table 4).

**Table 4:** The Pooled prevalence and ORs of respiratory viruses among under five years of age children in sub-Saharan Africa.

Viruses	Pooled prevalence among cases (%)	Pooled prevalence among controls (%)	OR[95 % CI]
Influenza A virus	4.24	1.06	3.49[2.50, 4.88]
Influenza B	1.07	0.41	2.06[1.26, 3.38]
Influenza C	0.53	0.69	0.85[0.43, 1.71]
Metapneumovirus	7.55	2.92	3.07[1.87, 5.06]
Bocavirus	11.45	11.96	1.05[0.75, 1.48]
Corona Virus	8.08	10.51	0.73[0.59, 0.89]
Parainfluenza virus	11.51	6.09	2.10[1.72, 2.56]
Adeno virus	12.44	9.06	1.39[1.20, 1.60]
Rhino virus	26.38	26.33	1.12[0.97, 1.30]

## 4 Discussion

This systematic review and meta-analysis reinforces RSV as an important cause of ALRI in young children in sub-Saharan Africa, and provides quantitative estimates of the absolute proportion of RSV–associated ALRI cases to which a viral cause can be attributed (OR 7.04; AFE 85.8 %; PAF 20.2). A previous global systematic review and meta–analysis in children under five years also supported the causal attribution of RSV (OR 9.79; AFE 90 %) [31]. Older adults with ALRIs were also more likely to have RSV when compared to asymptomatic or healthy controls (OR, 8.5 [95 % CI (3.9–18.5)] [32].

There is considerable global attention on RSV associated ALRI in young children and efforts to develop a RSV vaccine remain highly active. The susceptible populations for RSV infections are varied and include neonates, young children, pregnant women, and older adults. Induction of protective immune responses in each of these groups may require different vaccine types, adding a further challenge for vaccine development [33]. Generally, RSV prevention candidates that are in clinical development have used six different approaches: recombinant vector, subunit, particle-based, live attenuated, chimeric, and nucleic acid vaccines; and monoclonal antibodies [34]. The most promising vaccine candidates in infants and children have been LID ΔM2-2, MEDI M2-2, RSVcps2 and LID/ΔM2-2/1030s (live-attenuated) [35]. Based on our findings, effective vaccines would potentially prevent 20.2 % (PAF) of ALRIs in under five year's children in sub-Saharan Africa.

In sub-Saharan Africa region, where there are very limited viral infections diagnostic capacity, it is a common practice to empirically prescribe antibiotics to treat ALRIs s [36]. RSV is one of the important contributors to antimicrobial exposure among children. Effective vaccines could prevent antimicrobial prescribing and contribute to attenuation of antimicrobial resistance [[37], [38], [39]]. A randomized trial study demonstrated that administering an RSV vaccine to pregnant mothers reduced antimicrobial prescribing among their infants by 12.9 % over the first 3 months of life [40]. Therefore, prevention of RSV through vaccines could also help in fighting the current antimicrobial resistance challenge.

Considering the huge health and economic burden of RSV disease in sub-Saharan Africa, the potential interventions against RSV among children under 5 years are likely to be cost-effective. The RSV-associated disease burden among children in the 72 GAVI (The Global Alliance for Vaccines and Immunization) countries (most of them are sub-Saharan Africa countries) is estimated to be an average of 20.8 million cases, 1.8 million hospital admissions, 40 thousand deaths, 1.2 million discounted DALYs, and US$611 million discounted direct costs [41]. Out of the 49 Sub-Saharan Africa countries, 34 countries are among the least developed countries (LDCs), where approximately half of the population lives below the poverty line of "$1.25/day” [42]. Therefore, this virus is contributing a very high economic burden for the world poorest countries in sub-Saharan Africa.

This study was limited by the inclusion of only 7 countries (Botswana, Gambia, Kenya, Madagascar, Mali, South Africa and Zambia) out of 49 sub-Saharan Africa countries. We found a limited number of articles that fulfilled our inclusion criteria. More accurate estimates of the RSV burden in sub-Saharan Africa will require more active RSV surveillance and research programs that includes both ALRI and healthy children.

## 5 Conclusion

This systematic review and meta-analysis provides accurate and timely RSV disease burden estimates in young children of sub-Saharan Africa to inform future policies and interventions. In this systematic review and meta-analysis, we showed a high magnitude of RSV in <5 years children with a clear associations between RSV and ALRI hospitalization in young children in sub-Saharan Africa. Given the possibility of an RSV vaccine, this review provides useful baseline data for future studies assessing the interventions in Sub-Saharan Africa and early introduction of RSV vaccine once licensed.

## Data availability statement

Data included in article/supp. material/referenced in article.

## Funding

This research received no external funding.

## Institutional review board statement

Not applicable.

## Informed consent statement

Not applicable.

## CRediT authorship contribution statement

**Fiseha Wadilo:** Writing – original draft, Software, Methodology, Formal analysis, Data curation, Conceptualization. **Minyahil Tadesse Boltena:** Formal analysis, Data curation. **Rawliegh Howe:** Writing – review & editing, Supervision. **Fithamlak Bistegen Solomon:** Writing – original draft. **Adey Feleke:** Writing – review & editing, Supervision. **Tamrayehu Seyoum:** Data curation. **Andargachew Mulu:** Writing – review & editing, Conceptualization. **Adane Mihret:** Writing – review & editing, Conceptualization.

## Declaration of competing interest

The authors declare that they have no known competing financial interests or personal relationships that could have appeared to influence the work reported in this paper.
