# Supplementary material for: Burden of respiratory syncytial virus diseases among under 5 children in Sub-Saharan Africa: A systematic review and meta-analysis
Source: Heliyon. 2023 Nov 19;9(12):e22211. doi: 10.1016/j.heliyon.2023.e22211 (PMC10709164; doi:10.1016/j.heliyon.2023.e22211)
Supplement: Multimedia component 1 [file mmc1.docx]

**Supplementary tables**

| **Database** | **Query ID** | **Query** | **Results** |
| --- | --- | --- | --- |
| **PubMed** |  | (( "Respiratory Syncytial Virus Infections/diagnosis"[Mesh] OR "Respiratory Syncytial Virus Infections/epidemiology"[Mesh] OR "Respiratory Syncytial Virus Infections/etiology"[Mesh] OR "Respiratory Syncytial Virus Infections/virology"[Mesh] )) AND "Africa South of the Sahara"[Mesh] | 153 |
| **Google scholar** | QS1 | respiratory infections virus "case control" | 2,610 |
|  | QS2 | Respiratory Syncytial Virus "case control" | 364 |
| **EMBASAE** | QS1 | Respiratory Syncytial Virus | 113,792 |
|  | QS2 | 'africa'/exp OR 'angola'/exp OR 'benin'/exp OR 'botswana'/exp OR 'burkina faso'/exp OR 'burundi'/exp OR 'cameroon'/exp OR 'cape verde'/exp OR 'central african republic'/exp OR 'chad'/exp OR 'comoros'/exp OR 'congo'/exp OR 'cote d`ivoire'/exp OR 'djibouti'/exp OR 'equatorial guinea'/exp OR 'eritrea'/exp OR 'ethiopia'/exp OR 'gabon'/exp OR 'gambia'/exp OR 'ghana'/exp OR 'guinea'/exp OR 'guinea-bissau'/exp OR 'kenya'/exp OR 'lesotho'/exp OR 'liberia'/exp OR 'madagascar'/exp OR 'malawi'/exp OR 'mali'/exp OR 'mauritania'/exp OR 'mauritius'/exp OR 'mozambique'/exp OR 'namibia'/exp OR 'niger'/exp OR 'nigeria'/exp OR 'reunion'/exp OR 'rwanda'/exp OR 'sao tome and principe'/exp OR 'senegal'/exp OR 'seychelles'/exp OR 'sierra leone'/exp OR 'somalia'/exp OR 'south africa'/exp OR 'sudan'/exp OR 'south sudan'/exp OR 'eswatini'/exp OR 'tanzania'/exp OR 'togo'/exp OR 'uganda'/exp OR 'western sahara'/exp OR 'zambia'/exp OR 'zimbabwe'/exp | 141,022 |
|  | QS3 | #1 AND #2 | 423 |
| **SCOPUS** | QS1 | " Respiratory Syncytial Virus " OR "children" OR "acute respiratory tract infections" | [79,248](https://www-scopus-com.proxy.library.emory.edu/search/history/results.uri?origin=searchhistory&shid=1) |
|  | QS2 | "Angola" OR "Benin" OR "Botswana" OR "Burkina Faso" OR "Burundi" OR "Cape Verde" OR "Cameroon" OR "Central African Republic" OR "Chad" OR "Comoros" OR "Democratic Republic Congo" OR "Congo" OR "Cote D`Ivoire" OR "equatorial Guinea" OR "Eritrea" OR "Eswatini" OR "Ethiopia" OR "Gabon" OR "Gambia" OR "Ghana" OR "Guinea" OR "Guinea-Bissau" OR "Kenya" OR "Lesotho" OR "Liberia" OR "Madagascar" OR "Malawi" OR "Mali" OR "Mauritania" OR "Mauritius" OR "Mozambique" OR "Namibia" OR "Niger" OR "Nigeria" OR "Rwanda" OR "Sao tome and Principe" OR "Senegal" OR "Seychelles" OR "Sierra Leone" OR "Somalia" OR "South Africa" OR "South Sudan" OR "Sudan" OR "Tanzania" OR "Togo" OR "Uganda" OR "Zambia" OR "Zimbabwe" | [46,179](https://www-scopus-com.proxy.library.emory.edu/search/history/results.uri?origin=searchhistory&shid=2) |
|  | QS3 | #1 AND #2 | [296](https://www-scopus-com.proxy.library.emory.edu/search/history/results.uri?origin=searchhistory&shid=3) |
| **Web of science** | **Q1** | " Respiratory Syncytial Virus " OR "acute respiratory tract infections" OR “Pneumonia” | [36,283](https://www-webofscience-com.proxy.library.emory.edu/wos/woscc/summary/e7f6f8f0-1ad3-470c-af36-887bafbf32bf-48d97ee9/relevance/1) |
|  | **Q2** | TS=(Angola* OR Benin* OR Botswana* OR Burkina Faso* OR Burundi* OR Cape Verde* OR Cameroon* OR Central African Republic* OR Chad* OR Comoros* OR Democratic Republic Congo* OR Congo* OR Cote D`Ivoire* OR equatorial Guinea* OR Eritrea* OR Eswatini* OR Ethiopia* OR Gabon* OR Gambia* OR Ghana* OR Guinea* OR Guinea-Bissau* OR Kenya* OR Lesotho* OR Liberia* OR Madagascar* OR Malawi* OR Mali* OR Mauritania* OR Mauritius* OR Mozambique* OR Namibia* OR Niger* OR Nigeria* OR Rwanda* OR Sao tome and Principe* OR Senegal* OR Seychelles* OR Sierra Leone* OR Somalia* OR South Africa* OR South Sudan* OR Sudan* OR Tanzania* OR Togo* OR Uganda* OR Zambia* OR Zimbabwe*) | [202,536](https://www-webofscience-com.proxy.library.emory.edu/wos/woscc/summary/1c304a9b-07b1-494c-ae06-da8b3c0b4c06-48d990d2/relevance/1) |
|  | **Q3** | #1 AND #2 | [331](https://www-webofscience-com.proxy.library.emory.edu/wos/woscc/summary/631e9624-6387-4ce9-ae8f-8287726b3ca3-48d99afb/relevance/1) |
